# Modes of Competition: Adding and Removing Brown Trout in the Wild to Understand the Mechanisms of Density-Dependence

**DOI:** 10.1371/journal.pone.0062517

**Published:** 2013-05-02

**Authors:** Rasmus Kaspersson, Fredrik Sundström, Torgny Bohlin, Jörgen I. Johnsson

**Affiliations:** Department of Biological and Environmental Sciences, Animal Ecology, University of Gothenburg, Gothenburg, Sweden; Institut Pluridisciplinaire Hubert Curien, France

## Abstract

While the prevalence of density-dependence is well-established in population ecology, few field studies have investigated its underlying mechanisms and their relative population-level importance. Here, we address these issues, and more specifically, how differences in body-size influence population regulation. For this purpose, two experiments were performed in a small coastal stream on the Swedish west coast, using juvenile brown trout (*Salmo trutta*) as a study species. We manipulated densities of large and small individuals, and observed effects on survival, migration, condition and individual growth rate in a target group of intermediate-sized individuals. The generality of the response was investigated by reducing population densities below and increasing above the natural levels (removing and adding large and small individuals). Reducing the density (relaxing the intensity of competition) had no influence on the response variables, suggesting that stream productivity was not a limiting factor at natural population density. Addition of large individuals resulted in a negative density-dependent response, while no effect was detected when adding small individuals or when maintaining the natural population structure. We found that the density-dependent response was revealed as reduced growth rate rather than increased mortality and movement, an effect that may arise from exclusion to suboptimal habitats or increased stress levels among inferior individuals. Our findings confirm the notion of interference competition as the primary mode of competition in juvenile salmonids, and also show that the feedback-mechanisms of density-dependence are primarily acting when increasing densities above their natural levels.

## Introduction

Although negative density-dependence, through reduced survival and/or fecundity, is a well-established process for hampering population growth as resources become limited, the underlying mechanisms and their relative importance are less well known [1]. For example, intraspecific competition is likely to influence population regulation and dynamics differently depending on whether exploitation (depletion of resources through indirect interactions) or interference (restriction of resources through direct interactions) (sensu [2]) is the prevailing mode of competition (reviewed by [3], [4]). However, few studies have applied an experimental approach to investigate the relative population-level effects of interference and exploitation competition, and even fewer are performed in the field [5]–[8].

Among juvenile stream-living salmonids, interference through territoriality and dominance hierarchies is generally assumed to be the most prevalent mode of competition with strong density-dependent effects on mortality and migration, especially noticeable at the early stages after emergence [9]. The recognition that density-dependent growth rate may be most prevalent at low density (lower than required to elicit an interference response) [10], has led to the suggestion of exploitation, rather than interference competition, as the underlying mechanism [11], [12] (but see also [13]). If exploitation and interference competition indeed co-operate as density-dependent mechanisms in populations of stream-living salmonids, small individuals may have a competitive advantage under certain conditions and alter the resource acquisition of the remaining members (also larger individuals) of the population [14]. Hence, while individuals with a large body-size (in relation to the rest of the population) are likely to successfully defend a resource against smaller individuals at low density (i.e. through interference competition), such a pattern may shift to indirect exploitation competition, to the benefit of small-sized individuals, as the competitor density increases and/or as the size-difference between competitors diminishes.

For example, young (and small) individuals of planktivorous vendace (*Coregonus albula*) have competitive advantage over older (and larger) conspecifics, through lower metabolic requirements, with subsequent negative effects on growth rate and ultimately also on fecundity of the latter [15]. Indirect evidence for such a pattern among stream-living salmonids has been recently provided by Einum et al. [16], where even very low densities (between 0 and 1 individuals per m^2^) of salmon (*Salmo salar*) fry had negative effects on the growth of older cohorts. Suggested explanations for this response include shadow (filtering) competition where fry either depletes the downstream drift of invertebrates or intercepts the territories of older fish [17], as well as reduced benefits of territory defence at elevated densities (i.e. increased time spent fighting against fry) [18]. Studies correlating dominance behaviour of stream salmonids with performance in the field provides further support for the presence of alternative competitive strategies [19]–[21]. Höjesjö et al. [19] found that trout scored as non-aggressive in lab were able to grow as fast as dominant aggressive conspecifics when released into a heterogeneous natural stream environment, suggesting that difficulties in defending a resource will favour individuals with a more exploitative type of resource acquisition [22], [23].

Most knowledge concerning density-dependent regulation in stream-living salmonids is based on observational time-series data, making underlying mechanisms difficult to establish [13]. Moreover, while experiments manipulating densities of stream-living salmonids in field are rare, even fewer investigate density-dependence at or below natural population densities (but see e.g. [24], [25]). While population densities experimentally increased above carrying capacity may give valuable and important insights into the effects of stocking practices, the observed effects may not necessarily be applicable to natural conditions [26].

To better understand how body-size influence density-dependent population regulation, two field experiments were conducted on juvenile (1-yr old) sea-migratory brown trout (*Salmo trutta*). The competitive environments were altered by reducing (hereafter referred to as the Reduction experiment) and increasing (hereafter referred to as the Addition experiment) the number of large and small individuals and the density-dependent responses on growth, condition, survival and migration were evaluated in a target group of intermediate-sized individuals. Furthermore, based on suggestions in previous literature [10]–[12], we predicted that the competitive environments manipulated in the field experiments would differentiate in their density-dependent response, with reduction/addition of large individuals primarily influencing mortality and migration rate (through interference competition) and reduction/addition of small individuals primarily influencing individual growth rate (through exploitation competition) of the target group.

## Materials and Methods

### Ethics statement

This study was performed in accordance with Swedish animal welfare laws and was approved by the Gothenburg Ethical Committee (132–2005). Electro-fishing permits were obtained from the County Administrative Board Västra Götaland and all field work was approved by the private land owners. No protected species were collected during the field experiment.

### Study sites

The experiments were performed during 2006 (Reduction) and 2007 (Addition) in Jörlandaån (57°58′N; 11°55′E), a small stream 40 km north of Gothenburg on the Swedish west coast. Jörlandaån has a catchment area of 39 km^2^ and runs through deciduous forests with much vegetative overhang [27]. The stream is characterized by large seasonal changes in water discharge, usually with maximum flow in winter, and lowest flow during summer. The experimental sections are characterized by alternating pool-riffle habitats with mainly gravel-stone substrates and little macrophytic vegetation. The stream width and depth range between 2–4 m and 0.2–1 m, respectively. The dominant fish species is sea-migratory brown trout (>95% of all individual fish), which spend about two years in the stream before migrating to the sea (average population density in autumn is approximately 1 m^−2^ for young-of-the-year and yearling trout). Larger trout typically migrate sooner than smaller and size appears to be the main determinant of migration. After 1–2 years in the sea, adults return to their natal stream to breed. There is also a varying, but small, proportion of resident (non-migratory) trout that mature in the stream and they typically occupy the deeper pools. Predators are heron (*Ardea cinerea*), eel (*Anguilla anguilla*), larger resident brown trout, and mink (*Mustela vison*).

The experimental area was a stream stretch of approximately 1 km, within which three or four blocks (Reduction and Addition experiment, respectively) were distributed. Each block (replicate) consisted of three treatment units (stream sections) that were randomly distributed within the block and individually labelled on the stream bank:

Large individuals removed (Reduction experiment) or added (Addition experiment)

Small individuals removed (Reduction experiment) or added (Addition experiment)

Control, natural density and size-structure

The length of the stream sections ranged between 40 and 60 m, determined by the number of target fish (length required to capture approximately 30 target fish; see below). Each treatment section was isolated with a buffer zone of approximately 20 m in length with natural density and population size-structure.

### Experimental design

#### Reduction experiment

The population-level response of altered competition conditions was investigated by removing one third of the total biomass in two out of three experimental sections per block, either many small (lower third) or few large individuals (upper third), while keeping the natural density intact in the remaining control section (see Table S1 for the distribution of blocks and treatment sections). The response of the density manipulations was monitored in a target group of intermediate-sized individuals (middle third). Hence, the experimental design required a division of the total biomass in each treatment section into thirds (small, target (intermediate-sized) and large individuals) and in order to avoid sampling the fish twice (once for obtaining total biomass and biomass limits and once for sorting fish into respective group), this was achieved by a subsampling procedure (see below).

The experiment started 9–15 May 2006. Stream sections were sequentially electro-fished (LUGAB 1000, straight DC, 200-400V) beginning at the downstream end of the experimental site. All fish were captured and electro-fishing was paused after having collected approximately 30 individuals of intermediate size (target fish) (section length required to capture 30 target individuals ranged between 40–60 m). Collected fish were held in section-specific containers with aerated and continuously replaced stream water. The lower/middle/upper thirds of the biomass in each treatment section were estimated using a randomly selected sample of approximately 35 individuals from each section. Fish in the subsample were measured (fork length, to the nearest mm) and their individual body-weight was estimated using a length-weight relationship from previous population surveys at the same site and at the same time of year, in order to avoid anaesthetizing the fish and hence to reduce handling stress. The accumulated biomass of the subsample and the lower/middle/upper thirds were calculated in an Excel spread-sheet and the corresponding size-limits of the small group, the target group and the group of large fish were generated after sorting the subsample by body-length (see Sheet S1 and Fig. S1 for detailed information about this procedure).

The sampling procedure continued with all fish from each section being anaesthetized (2-phenoxyethanol, 0.5 ml/L), measured (to the nearest mm), weighed (to the nearest g) and placed in three separate containers according to the size-groups estimated from the subsample. Target fish (intermediate-sized individuals) were equipped with passive integrated transponders (PIT-tags, Trovan ID 100, 12 mm) for individual identification (Table 1).

**Table 1:** Initial number and biomass of trout size-groups in the Reduction experiment (2006).

Block	Treatment	Number of fish					Biomass (g)				
		Small	Target	Large	Tot.	Removed (%)^1^	Small	Target	Large	Tot.	Removed (%)^1^
**1**	**Small removed**	54	30	14	98	**54 (55)**	143.4	163.5	158.4	465.3	**143.4 (30.8)**
**1**	**Large removed**	42	38	25	107	**25 (23)**	96.7	149.9	179.1	425.7	**179.1 (42.1)**
**1**	**Control**	56	32	24	112	**-**	154	137.8	202.9	494.7	**-**
**2**	**Control**	45	47	11	103	**-**	93.3	196.2	118.7	408.2	**-**
**2**	**Large removed**	47	30	13	89	**13 (15)**	119.2	138.5	132.6	390.3	**132.6 (34.0)**
**2**	**Small removed**	45	27	19	91	**45 (49)**	116.7	122.3	163	402.0	**116.7 (29.0)**
**3**	**Large removed**	48	39	12	100	**12 (12)**	126.1	189.4	150.6	466.1	**150.6 (32.3)**
**3**	**Small removed**	40	42	9	91	**40 (44)**	128.2	229.7	116.8	474.7	**128.2 (27.0)**
**3**	**Control**	28	41	28	97	**-**	71.2	162.1	219.6	452.9	**-**
**Total**		**405**	**326**	**155**	**888**	**189 (32**±**7** 2 **)**	**1048.8**	**1489.4**	**1441.7**	**3979.9**	**850.6 (32.5**±**2.2** 2 **)**

1 number and biomass of removed fish. Percentage removed fish in relation to total section number and biomass is given within brackets and was used as a covariate in statistical analyses after arcsine(sqrt)-transformation

2 mean percentage removed fish (±SE)

After recuperating, the target group and a group of small, large or both (depending on treatment) were transferred back into the same stream section where they were captured, while removed small or large fish were released downstream of the experimental area. The release site was separated from the experimental area by a stream stretch of approximately 1.5 km consisting of low water level and poor habitat quality, in order to reduce the risk of homing.

**Figure 1:**
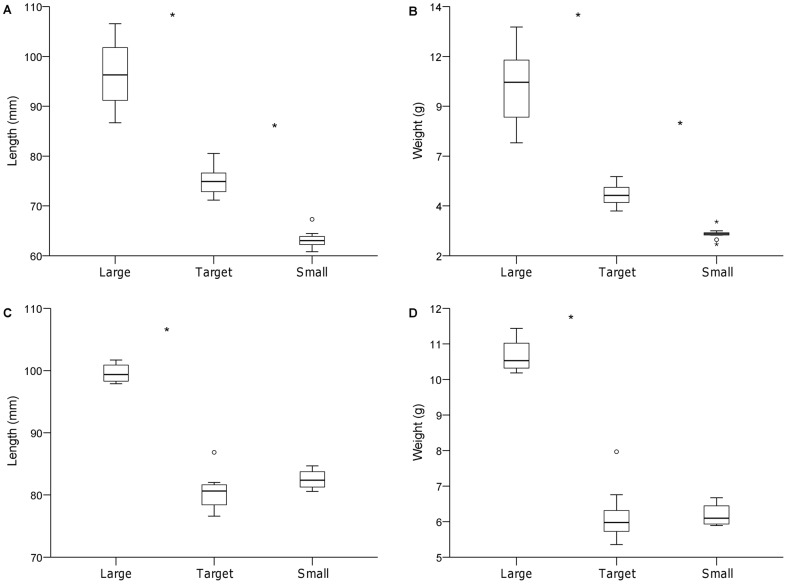
(A–D). Initial body-size of the three size-groups (small, intermediate (target) and large). Boxplots showing the initial length and weight at the Reduction experiment (A, B) (removed biomass) and the Addition experiment (C, D) (added biomass) with minimum and maximum (whiskers), first quartile, median and third quartile (box) and outliers (circles). Significant differences between groups are denoted by asterisks.

Estimated body-weights and size-limits of the three groups gave a relatively good fit when applied to the total data set (the biomass of the size-groups did not deviate greatly from 33% (one third) of the total section biomass). Biomass of removed large individuals was 36±3% (mean±SE of the total biomass) (which corresponded to 17±3% of the total number of fish), while 29±1.1% of the biomass was removed as small individuals (50±3% of the total number of fish) (Table 1). As expected, the three size-groups differed in weight (small: 2.6±0.1 g; target: 4.6±0.2 g; large: 10.0±0.7 g) and length (small: 63.0±0.8 mm; target: 75.4±1.1 mm; large: 96.2±2.4 mm) (mean±SE) (ANOVA, P<0.0001 both cases) (Fig. 1A–B) and there was no difference in initial size of target individuals between treatments (ANOVA, P>0.5 both cases).

Recaptures were performed on two occasions; one month after the start of the experiment (13–16 June 2006, R1) and in the autumn (25 September-4 October 2006, R2). A two-pass electro-fishing was performed at each recapture, starting 100 m downstream the first section and continuing 150 m upstream the uppermost section to include potential strayers from the experimental area. The capture location (block and section) was noted for each target individual and data on PIT-ID, length and weight was collected after anesthetising the fish. All individuals were released at the place of capture after recovering from the procedure.

#### Addition experiment

During the second year, different competitive environments were created by adding few large or many small individuals (from here on called supplementation fish) at a biomass equal to that of the target group in each experimental section, while keeping the natural densities intact in the remaining control section. The nine experimental sections of the preceding experiment were re-used during the Addition experiment and three additional sections were included to increase statistical power (see Table S1 for the distribution of blocks and treatment sections).

The experiment started 18–21 April 2007, when a two-pass electro-fishing was performed in each of the 12 experimental stream sections. Stream sections were sequentially electro-fished beginning at the downstream end of the experimental site, and captured fish were held in section-specific containers, with aerated and continuously replaced stream water. Fish were anaesthetized, weighed (to the nearest gram), measured (fork length, to the nearest mm) and individuals between 60–110 mm (the target group) were equipped with PIT-tags. This target group generally comprised a majority of the fish in the treatment sections (Table 2). The total biomass of the target group was calculated, and by using the maximum-likelihood solution for two successive removals according to Bohlin et al. [28], we could estimate the real section biomass and hence correct for fish not being captured during the electro-fishing (Table 2). After recuperating from the procedure, fish were transferred back into the same stream section as where they were captured.

**Table 2:** Initial number and biomass of trout size-groups in the Addition experiment (2007).

Block	Treatment	Number					Biomass (g)					
		Small	Target	Large	Tot.	Added (%)^1^	Small	Target	Large	Adj. target^3^	Tot.	Added (%)^1^
**1**	**Control**	13	50	8	71	**-**	20.0	305.0	220.8	311.4	545.8	**-**
**1**	**Small added**	6	77	39	122	**109 (89)**	11.2	416.8	1336.3	675.7	1764.3	**677.3 (38.4)**
**1**	**Large added**	13	57	33	103	**29 (28)**	23.1	257.4	1051.5	305.1	1332	**307.3 (23.1)**
**2**	**Small added**	16	54	20	90	**41 (46)**	26.8	270.1	666.2	269.2	963.1	**273.6 (28.4)**
**2**	**Large added**	4	28	38	70	**19 (27)**	7.2	180.2	2237.9	193.4	2425.3	**193.5 (8.0)**
**2**	**Control**	6	48	13	67	**-**	11.5	239.2	347.2	259.0	597.9	**-**
**3**	**Small added**	9	34	18	61	**37 (61)**	16.3	195.0	445.2	217.1	656.5	**221.2 (33.7)**
**3**	**Control**	10	49	23	82	**-**	18.4	287.9	527.5	322.0	833.8	**-**
**3**	**Large added**	4	36	12	52	**26 (50)**	8.9	243.3	240.5	289.0	492.7	**297.4 (60.4)**
**4**	**Small added**	9	29	17	55	**33 (60)**	16.4	173.5	408.5	189.2	598.4	**194.3 (32.5)**
**4**	**Large added**	1	60	39	100	**44 (44)**	2.0	324.9	1046.3	459.3	1373.2	**460.2 (33.5)**
**4**	**Control**	3	37	44	84	**-**	5.8	212.4	1123.8	249.2	1342.0	**-**
**Total**		**94**	**559**	**304**	**957**	**338 (51**±**7** 2 **)**	**167.6**	**3105.7**	**9651.7**	**3739.6**	**12925**	**2624.8 (32.2**±**5.2** 2 **)**

1 number and biomass of supplementation fish. Percentage added fish in relation to total section number and biomass is given within brackets and was used as a covariate in statistical analyses after arcsine(sqrt)-transformation

2 mean percentage added fish (±SE)

3 biomass adjusted for the catch probability

The supplementation fish were collected approximately one month after the experimental start-up (17 May 2007) at a site 3.5 km downstream the experimental area using electro-fishing. Immediately after capture, the fish were anaesthetized, measured (fork length, to the nearest mm), weighed (to the nearest gram), transported to the experimental area in aerated tanks and released in respective stream section.

In total, 220 small (63.9±9.2% of the total section density, mean±SE) and 118 large fish (37.3±5.7%) were added to the treatment sections (Table 2). The corresponding biomass of small and large supplementation fish was 1366 g (33.2±2.0% of the total section biomass) and 1258 g (31.2±11.0%) respectively. The group of large individuals had greater length (99.6±0.8 mm) and weight (10.7±0.3 g) than target individuals (length: 81.6±1.1; weight: 6.6±0.3) (mean±SE) (ANOVA, P<0.0001) but there was no difference in size between target individuals and the small group (length: 82.5±0.9; weight: 6.2±0.2) (P>0.1; Fig. 1C–D) and no difference in initial size of target individuals between treatment sections (ANOVA, P>0.5 both cases). Experimental sections were closed off with nets for the first 48 h after the addition to avoid fish being dispersed into adjacent areas.

Recaptures were performed on three occasions, two months after the start of the experiment (14–18 June 2007, R1), in the autumn (4–7 September 2007, R2) and after approximately one year (9–11 April 2008, R3). A two-pass electro-fishing was performed at each recapture, starting 100 m downstream the first section and continuing 150 m upstream the uppermost section to include potential strayers from the experimental area. The capture location (block and section) was noted for each target individual and data on PIT-ID, length and weight was collected after anesthetising the fish. All individuals were released at the place of capture after recovering from the procedure.

### Data treatment and analysis

Response variables were specific growth rate in weight (SGr_w_), difference in condition (C_diff_) from start to recapture, recapture rate (proportion of the released fish that were recaptured at the final sampling, proxy for survival) and movement (proportion of target fish recaptured within their home-section and home-block (section and block where they were released)). Specific growth rate was calculated as
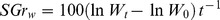
where *W_0_* is initial body-size and *W_t_* is body-size *t* days later. C_diff_ is the difference in residual value of the length-weight regression at the start and recapture, movement was analysed by assigning each recaptured individual a value of 1 (recaptured inside home-section or -block) and 0 (recaptured outside home-section or -block) and recapture rate was similarly analysed by assigning recaptured individuals a value of 1 and not recaptured individuals a value of 0.

Response variables of the Reduction experiment were calculated for the period between start of experiment (May) and final recapture (September-October, R2) (133-147 days). In the Addition experiment, response variables were calculated for the period between the start of the experiment (April) and final recapture (April subsequent year, R3) (354–358 days) but due to low recapture rates (84 individuals) these estimates were complemented by data for the period between start of experiment and the recapture in September (R2) (136–141 days).

Mean per section of the response variables (n = 9 (Reduction experiment) and n = 12 (Addition experiment)) were tested in an ANCOVA according to the following model:

Response variable  =  Treatment (fixed) + Block (random) + Initial length (covariate) + Proportion of total section biomass that was removed (Reduction experiment) or added (Addition experiment) (covariate)

with a step-wise removal of factors at P>0.1. Initial length refers to the mean length of target individuals in each section at the start of the experiment. Proportions were arcsine(sqrt)-transformed prior to analyses. SPSS (v.19) was used for all statistical analyses.

## Results

### Reduction experiment

A total of 175 individuals (54%) were recaptured at the final sampling in September-October (R2) (final densities of all captured trout are shown in Table S2). The catch probability for the target fish (calculated using the maximum-likelihood solution for two successive removals according to Bohlin et al. [28]) was estimated to 0.78 suggesting that 95% of the total target population was recaptured. Recapture rate was not affected by initial body-size or removed biomass and there was no difference between treatments (Table 3).

**Table 3:** Output of ANOVA testing the effect of Reduction and Addition of small and large trout on response variables in the target group. Period refers to the time during which response variables were analysed, from start of experiment (S) to Recapture 2 or 3 (R2 or R3).

Experiment	Period	Response variable	Block (*P*, F, MS, d.f.)	Treatment (*P*, F, MS, d.f.)	Length (*P*, F, MS, d.f.)	Removed/Added biomass (*P*, F, MS, d.f.)
**Reduction**	**S-R2**	**SGr_w_**	**0.044**, 7.547, 0.030, 2	0.119, 3.799, 0.015, 2	2	2
**Reduction**	**S-R2**	**C_diff_**	0.232, 2.154, 0.844, 2	0.133, 3.491, 1.368, 2	3	2
**Reduction**	**S-R2**	**Movement(Section)** 1	0.102, 4.265, 0.337, 2	0.350, 1.382, 0.109, 2	2	2
**Reduction**	**S-R2**	**Movement(Block)** 1	0.441, 1.012, 0.004, 2	0.666, 0.451, 0.002, 2	2	2
**Reduction**	**S-R2**	**Recapture rate** 1	0.181, 2.707, 0.026, 2	0.351, 1.377, 0.013, 2	2	2
**Addition**	**S-R2**	**SGr_w_**	**0.002**, 27.33, 0.021, 3	**0.002**, 28.60, 0.021, 2	**0.025**, 10.033, 0.008, 1	2
**Addition**	**S-R2**	**C_diff_**	0.231, 1.901, 9.610, 3	0.990, 0.010, 0.052, 2	3	2
**Addition**	**S-R2**	**Movement(Section)** 1	0.464, 0.977, 0.017, 3	0.553, 0.655, 0.011, 2	2	2
**Addition**	**S-R2**	**Movement(Block)** 1	0.581, 0.709, 0.059, 3	0.862, 0.152, 0.013, 2	2	2
**Addition**	**S-R3**	**SGr_w_**	**0.008**, 10.46, 0.003, 3	**0.001**, 25.71, 0.007, 2	2	2
**Addition**	**S-R3**	**C_diff_**	**0.028**, 6.270, 3.803, 3	0.685, 0.403, 3.803, 3	3	2
**Addition**	**S-R3**	**Movement(Block)** 1	0.715, 0.469, 0.209, 3	0.491, 0.804, 0.359, 2	2	2
**Addition**	**S-R3**	**Recapture rate** 1	**0.025**, 7.771, 0.027, 3	0.074, 4.596, 0.016, 2	**0.026**, 9.718, 0.034, 1	2

1 arcsine(sqrt) transformed proportions

2 removed (P>0.1)

3 not included in the model

While only 55% of the target fish were recaptured within their home-section, 88% stayed within their home-block. There was no difference in movement rate between treatments and no effect of initial body-size of the target fish (Table 3).

**Figure 2:**
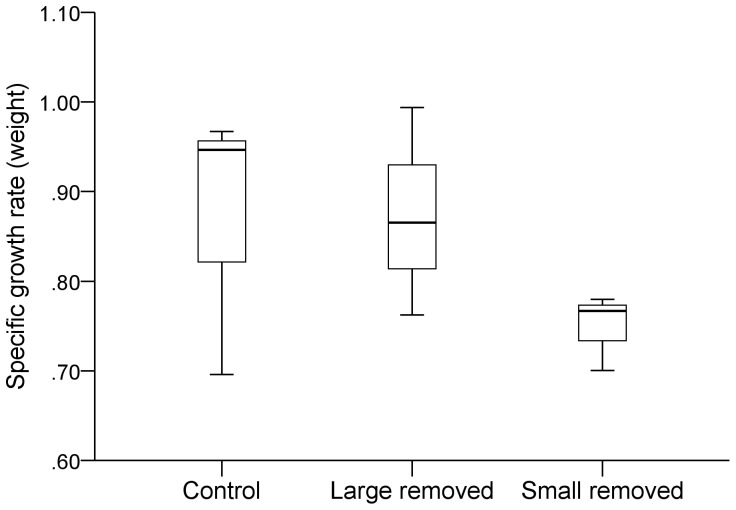
Growth of target fish during the Reduction experiment. Specific Growth rate in weight (SGr_w_, % growth day^−1^) of intermediate-sized target fish during the period May to September-October 2006, after removing large or small individuals. Boxplots show minimum and maximum (whiskers), first quartile, median and third quartile (box) and outliers (circles).

All recaptured target individuals maintained positive growth throughout the experimental period. While growth rate was different between blocks, no effects of initial body-size, removed biomass, or treatment were detected (Fig. 2; Table 3). Similarly, none of the model factors had a significant effect on the difference in condition residuals between the experiment start-up and the recapture.

### Addition experiment

A total of 81 individuals (14%) were recaptured during the final sampling (April 2008, R3), with no effect of treatment or added biomass (Table 3) (final densities of all captured trout are shown in Table S2). Due to the low recapture rate, movement, growth rate and condition were analyzed at both the final recapture and at the recapture before winter (September 2007: 244 individuals, 44%) (70 individuals were captured at both occasions). The catch probability for the target fish was estimated to 0.37 and 0.72 suggesting that 60% and 94% of the total target population was recaptured the final recapture and at the recapture before winter, respectively.

Most target fish moved only short distances, with 75% being recaptured within their home-section and 89% within their home-block before winter (September, R2) while no target individuals were recaptured in their home-section after winter (April, R3) but 57% within their home block. There was no difference in movement rate between treatments at either of the occasions (Table 3).

**Figure 3:**
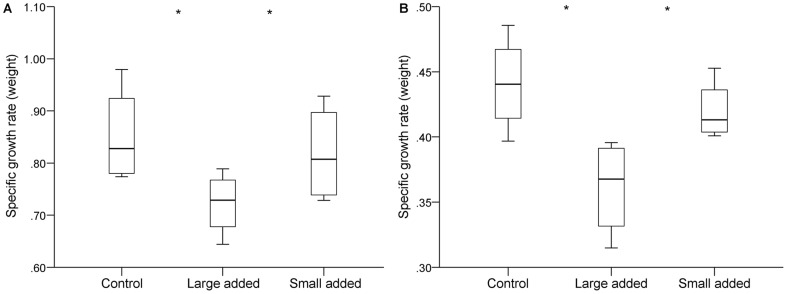
(A–B). Growth of target fish during the Addition experiment. Specific Growth rate in weight (SGr_w_, % growth day^−1^) of intermediate-sized target fish during the periods April to September 2007 (A) and April 2007 to April 2008 (B), after adding large or small individuals. Boxplots show minimum and maximum (whiskers), first quartile, median and third quartile (box) and outliers (circles). Significant differences between groups are denoted by asterisks.

All recaptured target individuals maintained positive growth throughout the sampling period. Growth rate was significantly affected by block and treatment at both recapture occasions (R2 and R3) (Table 3), with target individuals growing slower in treatment sections with large individuals added compared to control sections (natural density) and sections with small individuals added (Fig. 3A–B). As for the Reduction experiment, treatment had no effect on the difference in condition residuals between the start of the experiment and the recapture (Table 3).

## Discussion

To our knowledge, these are the first field studies to explore the mechanisms of density-dependence in a stream-living salmonid population using a fully experimental approach. We tested for differential effects of adding and removing large and small individuals on a group of intermediate-sized individuals. Most field evidence of density-dependence in salmonids is derived from populations that are experimentally increased by the addition of supplementation fish, either hatchery-reared alone (e.g. [29]–[31]) or together with translocated wild stocks (e.g. [27], [32], [33]). Hence, in this study, we tested for the generality of such an experimental design by reducing below as well as increasing above the natural population density (the population carrying capacity).

Reducing the population density below the natural level by removing few large and several small individuals had no feedback-effects on the performance of intermediate-sized target fish. This finding contrasts the response of recent studies using a similar experimental approach but investigating competition effects between, rather than within, cohorts [24], [25].

The high productivity of the stream used in this experiment may be one explanation for the lack of response. If resource availability was in excess at the natural population density, i.e. if the natural population density was below carrying capacity, we would indeed expect the effect of density reduction to be less apparent. Such a scenario would also appear if the population density is pushed below carrying capacity through high mortality at the early critical phase after emergence, as suggested in the extensive work by Elliott [9] in Black Brows Beck, rather than remaining at carrying capacity throughout development, as suggested by the self-thinning models (e.g. [34], [35]). An additional explanation may be the use of competitive strategies that enables intermediate-sized (target) individuals to maintain their level of resource acquisition at both tested densities (natural and low), but such a mechanism is difficult to substantiate with the current experimental design.

Increasing the natural population density by adding few large and several small individuals gave rise to a negative feedback-effect. Target individuals in treatment sections, to which large fish were added, experienced a significantly slower growth rate whereas no such effect was detected in control sections (natural size-structure) or in sections with an increased number of small fish. The reduced growth rate was revealed at both tested time periods, from the experimental start-up to the autumn sample as well as to the spring sample, one year later. This finding suggests that the body-size of the introduced fish relative to that of the target group was more important for the performance of intermediate-sized target individuals, than population density per se. Such a response would be expected if feeding territories, rather than food itself, is the main limiting resource, with few large individuals being able to monopolize high-quality areas of the stream habitat [36].

The observed reduction of individual growth rate disagrees with the prediction that larger individuals (through interference competition) affect primarily mortality and movement. Several studies, especially the extensive observational data by Elliott [9], report intense density-dependent mortality of younger stages of brown trout. However, experimental field studies on similar-aged individuals as the ones used here [27], [30], corroborate a density-dependent reduction in growth rate, suggesting that the response may be regulated by an ontogenetic shift in the sensitivity to starvation [29]. Accordingly, low fat reserves results in elevated density-dependent mortality through starvation as the fry competes for feeding territories early after emergence. Older cohorts, on the other hand, have greater energy reserves, hence explaining the density-dependent effect on growth rate rather than mortality.

Although not specifically investigated here, several mechanisms may explain the observed reduction in growth rate among the target fish. Size-dependent habitat use is frequently reported within (e.g. [37]) as well as between salmonid cohorts (e.g. [25]), and it is likely that the availability of food items is negatively affected if target individuals are excluded to territories of lower quality by larger (and more dominant) supplementation fish. However, a reduction in food availability may also occur independent of habitat use if large individuals, as a consequence of their greater diet niche width [38], deplete the supply of small-sized food items favoured by the target fish. Subdominant individuals are generally assumed to be more sensitive to lowered food intake rate than dominant individuals, due to their higher energy expenditure [39], and such responses are therefore likely to translate rapidly into negative somatic effects (e.g. growth rate). In addition to reducing food availability, adding large dominant individuals may also have indirect effects by influencing the activity pattern of the target fish [40], forcing them to forage at less preferred hours of the day [41] and by increasing their stress levels [42].

In accordance with the findings of previous studies (e.g. [18], [43], [44]), we predicted that the competitive advantage of large body-size would decrease as the group-size increases. Hence, the addition of several small-sized individuals was predicted to make resource defence (i.e. interference) uneconomical and instead induce exploitation competition as the primary competition mode. Such shifts of competitive strategies have been observed in the lab [45] as well as under hatchery conditions [46], where high population densities and/or unpredictable resource distribution in time and space, makes resource monopolization unsustainable and reduces the level of aggression. Thus, the lack of a negative feedback response in this study suggests that the target group could successfully defend their feeding habitats, despite the addition of several small-sized supplementation fish, possibly through their prior residency in the stream habitat. Target individuals were returned to their respective stream section after tagging since random distribution within the experimental site was expected to induce high levels of homing and hence, disturb the experimental density manipulations [47]. As a result, and in agreement with similar studies [24], [27], [30], movement of target fish was restricted to areas adjacent to the release sites. The disadvantage of returning fish to their natal site is, however, that the prior residency of the target group may have inferred benefits [48] that enabled them to successfully defend resources at higher densities than expected if both groups were released into a novel stream area. This is in accordance with Johnsson et al. [49], who showed that juvenile brown trout intruders needed a size-advantage of 30% to overcome the benefits of prior residency in dyadic competitions. Hence, the same mechanism would also explain the negative feedback revealed when adding few large individuals, suggesting that their 22% size-advantage was sufficient to overrule the benefit of prior residency of the target group.

In conclusion, our results show that addition of few large individuals results in a negative feedback-response on the growth rate of intermediate-sized target individuals. We argue that this is likely an effect of interference competition, where the larger (and supposedly also more dominant) supplementation fish exclude target individuals from favourable feeding habitats. For organisms with indeterminate growth, such as fishes, a reduction in growth rate can have substantial life-history consequences, ultimately by affecting fecundity [50] and capacity to withstand harsh winter conditions [51], but in the case of sea-migratory salmonids, also for determining life-history tactics, timing of smoltification and time spent at sea (reviewed by [52]). The absence of a positive feedback-effect when removing small or large individuals (possibly relaxing interference and exploitation competition) suggests that the high productivity of the stream used in this experiment enabled the target fish to maintain growth independent of the tested population densities. An interpretation of our findings is that interference competition is the primary competition mode in juvenile salmonids, and that the feedback-mechanisms of density-dependence are primarily acting when increasing densities above their natural levels.

## Supporting Information

Figure S1 Length-weight relationships. Comparisons of the actual length-weight relationship (all captured individuals) (blue dots) (y = 6E−06x^3.1100^) with an estimation based on data from previous years of electro-fishing surveys (red dots) (y = 6E−06x^3.1258^).
(DOCX)

Sheet S1 Procedure for establishing size-limits during the Reduction experiment. A subsample of 35 individuals from each experimental stream section was collected to estimate the size-limits of the three size-groups (small, intermediate (target) and large) and applied to the total stock of each section.
(XLSX)

Table S1 Distribution of treatment sections and blocks within the experimental site. The reduction experiment comprised three blocks (replicates) while four blocks were used during the addition experiment.
(DOCX)

Table S2 Final densities. Average densities (individuals 100 m^−2^) of tagged and untagged (1 yr and older) fish at the final recapture of the Reduction experiment (September-October 2006) and the Addition experiment (April 2008), respectively.
(DOCX)

## References

[1] Tilman D (1987) The importance of the mechanisms of interspecific competition. Am Nat 129: 769–774 doi:10.1086/284672.

[2] Keddy PA (2001) Competition. Dordrecht: Kluwer Academic Publishers. 552 p.

[3] Hart DD (1987) Experimental studies of exploitative competition in a grazing stream insect. Oecologia 73: 41–47 doi:10.1007/bf00376975. 28311403 10.1007/BF00376975

[4] Scharf I, Filin I, Ovadia O (2008) An experimental design and a statistical analysis separating interference from exploitation competition. Popul Ecol 50: 319–324 doi:10.1007/s10144-008-0081-9.

[5] Davey AJH, Doncaster CP, Jones OD (2009) Distinguishing between interference and exploitation competition for shelter in a mobile fish population. Env Model Assess 14: 555–562 doi:10.1007/s10666-008-9171-5.

[6] Davey AJH, Turner GF, Hawkins SJ, Doncaster CP (2006) Mechanisms of density dependence in stream fish: exploitation competition for food reduces growth of adult European bullheads (Cottus gobio). Can J Fish Aquat Sci 63: 597–606 doi:10.1139/f05-246.

[7] Eccard JA, Ylonen H (2002) Direct interference or indirect exploitation? An experimental study of fitness costs of interspecific competition in voles. Oikos 99: 580–590.

[8] Cameron TC, Wearing HJ, Rohani P, Sait SM (2007) Two-species asymmetric competition: Effects of age structure on intra- and interspecific interactions. J Anim Ecol 76: 83–93. 17184356 10.1111/j.1365-2656.2006.01185.x

[9] Elliott JM (1994) Quantitative ecology and the brown trout. Oxford: Oxford University Press. 286 p.

[10] Jenkins TM, Diehl S, Kratz KW, Cooper SD (1999) Effects of population density on individual growth of brown trout in streams. Ecology 80: 941–956 doi:10.1890/0012-9658(1999)080[0941:EOPDOI]2.0.CO;2.

[11] Imre I, Grant JWA, Cunjak RA (2005) Density-dependent growth of young-of-the-year Atlantic salmon *Salmo salar* in Catamaran Brook, New Brunswick. J Anim Ecol 74: 508–516 doi:10.1111/j.1365-2656.2005.00949.x.

[12] Grant JWA, Imre I (2005) Patterns of density-dependent growth in juvenile stream-dwelling salmonids. J Fish Biol 67: 100–110 doi:10.1111/j.0022-1112.2005.00916.x.

[13] Ward DM, Nislow KH, Armstrong JD, Einum S, Folt CL (2007) Is the shape of the density-growth relationship for stream salmonids evidence for exploitative rather than interference competition? J Anim Ecol 76: 135–138 doi:10.1111/j.1365-2656.2006.01169.x. 17184361 10.1111/j.1365-2656.2006.01169.x

[14] Persson L (1985) Asymmetrical competition: Are larger animals competitively superior? Am Nat 126: 261–266.

[15] Hamrin SF, Persson L (1986) Asymmetrical competition between age classes as a factor causing population oscillations in an obligate planktivorous fish species. Oikos 47: 223–232.

[16] Einum S, Nislow KH, McKelvey S, Armstrong JD (2011) The spatial scale of competition from recruits on an older cohort in Atlantic salmon. Oecologia 167: 1017–1025 doi:10.1007/s00442-011-2055-4. 21710118 10.1007/s00442-011-2055-4 PMC3213340

[17] Elliott JM (2002) Shadow competition in wild juvenile sea-trout. J Fish Biol 61: 1268–1281 doi:10.1006/jfbi.2002.2154.

[18] Kaspersson R, Höjesjö J, Pedersen S (2010) Effects of density on foraging success and aggression in age-structured groups of brown trout. Anim Behav 79: 709–715 doi:10.1016/j.anbehav.2009.12.025.

[19] Höjesjö J, Johnsson J, Bohlin T (2004) Habitat complexity reduces the growth of aggressive and dominant brown trout (*Salmo trutta*) relative to subordinates. Behav Ecol Sociobiol 56: 286–289 doi:10.1007/s00265-004-0784-7.

[20] Harwood AJ, Armstrong JD, Metcalfe NB, Griffiths SW (2003) Does dominance status correlate with growth in wild stream-dwelling Atlantic salmon (Salmo salar)? Behav Ecol 14: 902–908 doi:10.1093/beheco/arg080.

[21] Puckett KJ, Dill LM (1985) The energetics of feeding territoriality in juvenile Coho salmon (Oncorhynchus kisutch). Behaviour 92: 97–111 doi:10.1163/156853985x00398.

[22] Grant JWA, Noakes DLG (1988) Aggressiveness and foraging mode of young-of-the-year Brook charr, *Salvelinus fontinalis* (Pisces, Salmoniidae). Behav Ecol Sociobiol 22: 435–445 doi:10.1007/bf00294982.

[23] Weir LK, Grant JWA (2004) The causes of resource monopolization: Interaction between resource dispersion and mode of competition. Ethology 110: 63–74 doi:10.1046/j.1439-0310.2003.00948.x.

[24] Kaspersson R, Höjesjö J (2009) Density-dependent growth rate in an age-structured population: a field study on stream-dwelling brown trout *Salmo trutta* . J Fish Biol 74: 2196–2215 doi:10.1111/j.1095-8649.2009.02227.x. 20735548 10.1111/j.1095-8649.2009.02227.x

[25] Kaspersson R, Höjesjö J, Bohlin T (2012) Habitat exclusion and reduced growth: a field experiment on the effects of inter-cohort competition in young-of-the-year brown trout. Oecologia 169: 733–742 doi:10.1007/s00442-012-2248-5. 22271199 10.1007/s00442-012-2248-5

[26] Brännas E, Jonsson S, Brännäs K (2004) Density-dependent effects of prior residence and behavioural strategy on growth of stocked brown trout (Salmo trutta). Can J Zool 82: 1638–1646 doi:10.1139/z04-147.

[27] Bohlin T, Sundström LF, Johnsson JI, Höjesjö J, Pettersson J (2002) Density-dependent growth in brown trout: Effects of introducing wild and hatchery fish. J Anim Ecol 71: 683–692 doi:10.1046/j.1365-2656.2002.00631.x.

[28] Bohlin T, Hamrin S, Heggberget TG, Rasmussen G, Saltveit SJ (1989) Electrofishing: Theory and practice with special emphasis on salmonids. Hydrobiologia 173: 9–43.

[29] Einum S, Sundt-Hansen L, Nislow KH (2006) The partitioning of density-dependent dispersal, growth and survival throughout ontogeny in a highly fecund organism. Oikos 113: 489–496 doi:10.1111/j.2006.0030-1299.14806.x.

[30] Sundström LF, Bohlin T, Johnsson JI (2004) Density-dependent growth in hatchery-reared brown trout released into a natural stream. J Fish Biol 65: 1385–1391 doi:10.1111/j.1095-8649.2004.00537.x.

[31] Baer J, Brinker A (2008) Are growth and recapture of hatchery-reared and resident brown trout (Salmo trutta L.) density dependent after stocking? Ecol Freshw Fish 17: 455–464 doi:10.1111/j.1600-0633.2008.00297.x.

[32] Berg S, Jorgensen J (1991) Stocking experiments with 0+ and 1+ trout parr, Salmo trutta L., of wild and hatchery origin. 1. Post-stocking mortality and smolt yield. J Fish Biol 39: 151–169 doi:10.1111/j.1095-8649.1991.tb04353.x.

[33] Jorgensen J, Berg S (1991) Stocking experiments with 0+ and 1+ trout parr, Salmo trutta L., of wild and hatchery origin. 2. Post-stocking movements. J Fish Biol 39: 171–180 doi:10.1111/j.1095-8649.1991.tb04354.x.

[34] Bohlin T, Dellefors C, Faremo U, Johlander A (1994) The energetic equivalence hypothesis and the relation between population density and body-size in stream-living salmonids. Am Nat 143: 478–493.

[35] Armstrong JD, Nislow KH (2006) Critical habitat during the transition from maternal provisioning in freshwater fish, with emphasis on Atlantic salmon (*Salmo salar*) and brown trout (*Salmo trutta*). J Zool 269: 403–413 doi:10.1111/j.1469-7998.2006.00157.x.

[36] Nakano S (1995) Individual differences in resource use, growth and emigration under the influence of a dominance hierarchy in fluvial red-spotted masu salmon in a natural habitat. J Anim Ecol 64: 75–84.

[37] Bremset G, Berg OK (1999) Three-dimensional microhabitat use by young pool-dwelling Atlantic salmon and brown trout. Anim Behav 58: 1047–1059 doi:10.1006/anbe.1999.1218. 10564607 10.1006/anbe.1999.1218

[38] Hubert WA, Harris DD, Rhodes HA (1993) Variation in the summer diet of age-0 brown trout in a regulated mountain stream. Hydrobiologia 259: 179–185 doi:10.1007/bf00006597.

[39] Metcalfe NB (1986) Intraspecific variation in competitive ability and food-intake in salmonids: consequences for energy budgets and growth rates. J Fish Biol 28: 525–531 doi:10.1111/j.1095-8649.1986.tb05190.x.

[40] Abbott JC, Dill LM (1989) The relative growth of dominant and subordinate juvenile steelhead trout (Salmo gairdneri) fed equal rations. Behaviour 108: 104–113 doi:10.1163/156853989X00079.

[41] Alanärä A, Burns MD, Metcalfe NB (2001) Intraspecific resource partitioning in brown trout: The temporal distribution of foraging is determined by social rank. J Anim Ecol 70: 980–986 doi:10.1046/j.0021-8790.2001.00550.x.

[42] Ejike C, Schreck CB (1980) Stress and social hierarchy rank in coho salmon. Trans Am Fish Soc 109: 423–426 doi:10.1577/1548-8659(1980)109<423:sashri>2.0.co;2.

[43] Kim JW, Grant JWA (2007) Effects of patch shape and group size on the effectiveness of defence by juvenile convict cichlids. Anim Behav 73: 275–280 doi:10.1016/j.anbehav.2006.08.003.

[44] Pettersson J, Johnsson JI, Bohlin T (1996) The competitive advantage of large body size declines with increasing group size in rainbow trout. J Fish Biol 49: 370–372 doi:10.1111/j.1095-8649.1996.tb00033.x.

[45] Brännäs E, Jonsson S, Brännäs K (2004) Density-dependent effects of prior residence and behavioural strategy on growth of stocked brown trout (Salmo trutta). Can J Zool 82: 1638–1646 doi:10.1139/z04-147.

[46] Alanärä A, Brännäs E (1996) Dominance in demand-feeding behaviour in Arctic charr and rainbow trout: The effect of stocking density. J Fish Biol 48: 242–254 doi:10.1111/j.1095-8649.1996.tb01116.x.

[47] Huntingford FA, Braithwaite VA, Armstrong JD, Aird D, Joiner P (1998) Homing in juvenile salmon in response to imposed and spontaneous displacement: experiments in an artificial stream. J Fish Biol 53: 847–852 doi:10.1111/j.1095-8649.1998.tb01838.x.

[48] Huntingford F, Turner A (1987) Animal conflict. London: Chapman and Hall. 448 p.

[49] Johnsson JI, Nöbbelin F, Bohlin T (1999) Territorial competition among wild brown trout fry: Effects of ownership and body size. J Fish Biol 54: 469–472 doi:10.1111/j.1095-8649.1999.tb00846.x.

[50] Jonsson N, Jonsson B, Fleming IA (1996) Does early growth cause a phenotypically plastic response in egg production of Atlantic Salmon? Funct Ecol 10: 89–96 doi:10.2307/2390266.

[51] Oliver JD, Holeton GF, Chua KE (1979) Overwinter mortality of fingerling smallmouth bass in relation to size, relative energy stores, and environmental temperature. Trans Am Fish Soc 108: 130–136 doi:10.1577/1548-8659(1979)108<130:omofsb>2.0.co;2.

[52] Klemetsen A, Amundsen PA, Dempson JB, Jonsson B, Jonsson N, et al (2003) Atlantic salmon *Salmo salar* L., brown trout *Salmo trutta* L. and Arctic charr *Salvelinus alpinus* (L.): A review of aspects of their life histories. Ecol Freshw Fish 12: 1–59 doi:10.1034/j.1600-0633.2003.00010.x.

