# Supplementary material for: Modes of Competition: Adding and Removing Brown Trout in the Wild to Understand the Mechanisms of Density-Dependence
Source: PLoS One. 2013 May 2;8(5):e62517. doi: 10.1371/journal.pone.0062517 (PMC3642212; doi:10.1371/journal.pone.0062517)
Supplement: Figure S1 — Length-weight relationships. Comparisons of the actual length-weight relationship (all captured individuals) (blue dots) (y = 6E−06x3.1100) with an estimation based on data from previous years of electro-fishing surveys (red dots) (y = 6E−06x3.1258). (DOCX) [file pone.0062517.s001.docx]

Modes of competition: Adding and removing brown trout in the wild to understand the mechanisms of density-dependence

Rasmus Kaspersson, Fredrik Sundström, Torgny Bohlin, Jörgen I. Johnsson

**
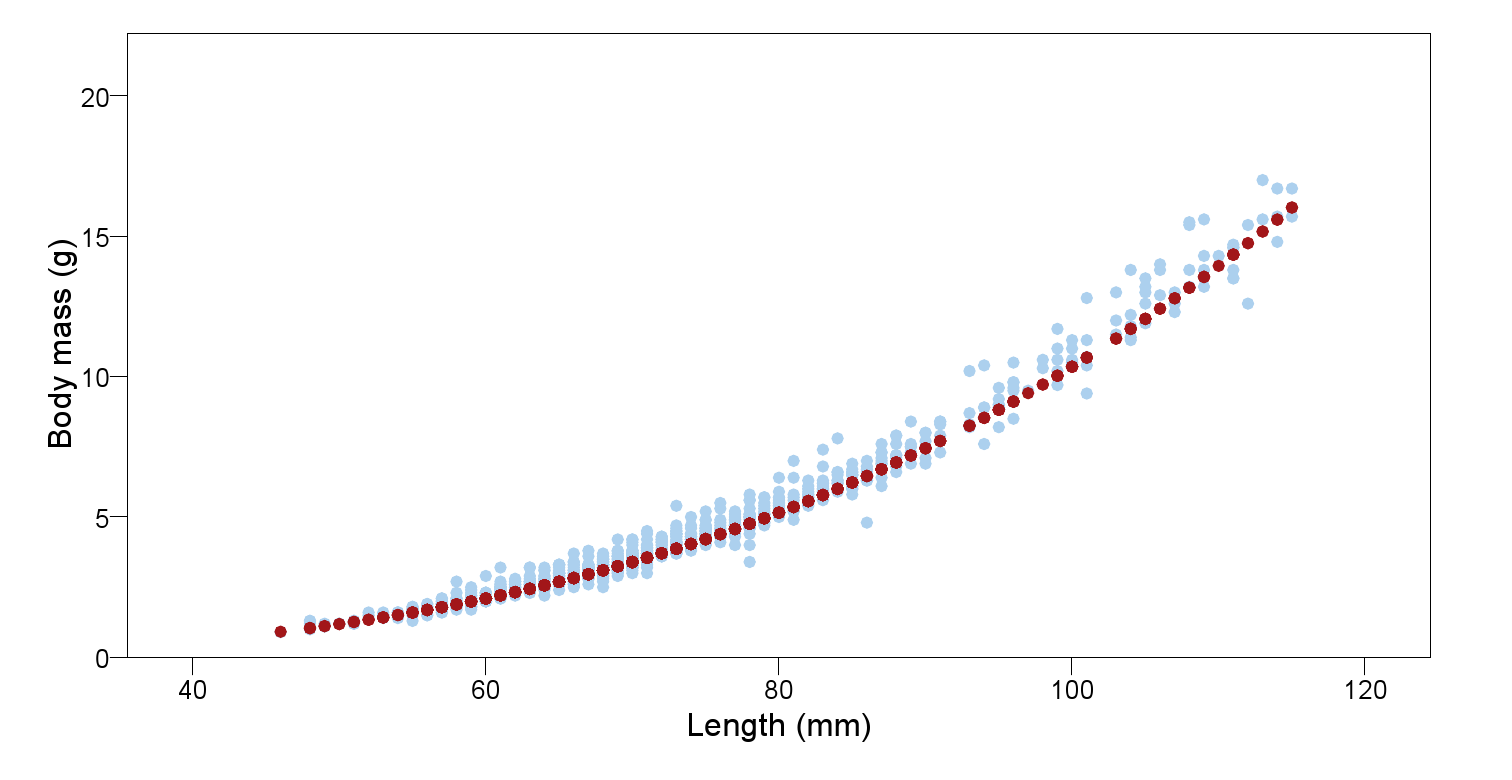
Figure S1.** **Length-weight relationships.** Comparisons of the actual length-weight relationship (all captured individuals) (blue dots) (y = 6E-06x^3.1100^) with an estimation based on data from previous years of electro-fishing surveys (red dots) (y = 6E-06x^3.1258^).
