# Supplementary material for: Modes of Competition: Adding and Removing Brown Trout in the Wild to Understand the Mechanisms of Density-Dependence
Source: PLoS One. 2013 May 2;8(5):e62517. doi: 10.1371/journal.pone.0062517 (PMC3642212; doi:10.1371/journal.pone.0062517)
Supplement: Table S1 — Distribution of treatment sections and blocks within the experimental site. The reduction experiment comprised three blocks (replicates) while four blocks were used during the addition experiment. (DOCX) [file pone.0062517.s003.docx]

Modes of competition: Adding and removing brown trout in the wild to understand the mechanisms of density-dependence

Rasmus Kaspersson, Fredrik Sundström, Torgny Bohlin, Jörgen I. Johnsson

**Table S1. Distribution of treatment sections and blocks within the experimental site.** The reduction experiment comprised three blocks (replicates) while four blocks were used during the addition experiment.

| **Direction of flow** | **Reduction experiment (2006)** | | | **Addition experiment (2007)** | | |
| --- | --- | --- | --- | --- | --- | --- |
|  | **Block** | **Section** | **Treatment** | **Block** | **Section** | **Treatment** |
| *Upstream* |  |  |  | 4 | 11 | Control |
|  |  |  |  | 4 | 10 | Large added |
|  | 3 | 9 | Control | 4 | 9 | Small added |
|  | 3 | 8 | Small removed | 3 | 8 | Large added |
|  | 3 | 7 | Large removed | 3 | 7 | Control |
|  | 2 | 6 | Small removed | 3 | 6 | Small added |
|  | 2 | 5 | Large removed | 2 | 5 | Control |
|  | 2 | 4 | Control | 2 | 4 | Large added |
|  | 1 | 3 | Control | 2 | 3 | Small added |
|  | 1 | 2 | Large removed | 1 | 2 | Large added |
|  | 1 | 1 | Small removed | 1 | 1 | Small added |
| *Downstream* |  |  |  | 1 | 0 | Control |
