# Supplementary material for: Modes of Competition: Adding and Removing Brown Trout in the Wild to Understand the Mechanisms of Density-Dependence
Source: PLoS One. 2013 May 2;8(5):e62517. doi: 10.1371/journal.pone.0062517 (PMC3642212; doi:10.1371/journal.pone.0062517)
Supplement: Table S2 — Final densities. Average densities (individuals 100 m−2) of tagged and untagged (1 yr and older) fish at the final recapture of the Reduction experiment (September-October 2006) and the Addition experiment (April 2008), respectively. (DOCX) [file pone.0062517.s004.docx]

Modes of competition: Adding and removing brown trout in the wild to understand the mechanisms of density-dependence

Rasmus Kaspersson, Fredrik Sundström, Torgny Bohlin, Jörgen I. Johnsson

**Table S2. Final densities.** Average densities (individuals 100 m^-2^) of tagged and untagged (1 yr and older) trout at the final recapture of the Reduction experiment (September-October 2006) and the Addition experiment (April 2008), respectively.

| **Reduction experiment (2006)** | | **Addition experiment (2007)** | |
| --- | --- | --- | --- |
| **Treatment** | **Final density (±SE)** | **Treatment** | **Final density (±SE)** |
| Control (3 replicates) | 136 ± 38 | Control (4 replicates) | 29 ± 12 |
| Large removed (3 replicates) | 87 ± 4 | Large added (4 replicates) | 41 ± 9 |
| Small removed (3 replicates) | 88 ± 8 | Small added (4 replicates) | 37 ± 4 |
